# Factors associated with delayed initiation of breast cancer treatment at an oncology referral center in Juiz de Fora, Minas Gerais state, from 2010 to 2019: a cohort study

**DOI:** 10.1590/S2237-96222024v33e20231177.en

**Published:** 2024-08-23

**Authors:** Fernanda de Paula Ciribelli da Silva, Mirian Carvalho Souza, Neilane Bertoni

**Affiliations:** 1 Associação Feminina de Prevenção e Combate ao Câncer de Juiz de Fora, Juiz de Fora, MG, Brazil; 2 Instituto Nacional de Câncer, Programa de Pós-Graduação em Saúde Coletiva e Controle do Câncer, Rio de Janeiro, RJ, Brazil

**Keywords:** Neoplasia de Mama, Hora de Iniciar el Tratamiento, Salud Pública, Estudio Observacional, Estudios de Cohorte, Breast Cancer, Time to Treatment Initiation, Public Health, Observational Study, Cohort Studies

## Abstract

**Objectives:**

To analyze factors associated with delayed initiation of breast cancer treatment at an oncology referral center in Juiz de Fora, Minas Gerais state, between 2010 and 2019.

**Methods:**

This was a cohort study using data from the Hospital-based Cancer Registry. The probability of not starting treatment within 60 days, in accordance with Brazilian law, was estimated using Kaplan-Meier, method and its association with the factors studied was assessed using the Cox model, presenting hazard ratios (HR) and respective 95% confidence intervals (95%CI).

**Results:**

Among the 911 participants, the probability of delayed treatment initiation was 18.8% (95%CI 16.4;21.5). Those who underwent treatment at a health service other than the one where the cancer was diagnosed had a significantly higher risk (HR: 3.49; 95%CI 3.00;4.07).

**Conclusion:**

Receiving a diagnosis and treatment at the same institution may help reduce waiting time to initiate cancer treatment.

## Study contributions

**Main results:**

The probability of study participants not initiating treatment within 60 days was 18.8%. Undergoing treatment at a healthcare service other than the one where the diagnosis was made was the main factor associated with delay.

**Implications for services:**

Organizing healthcare services based on strategies that optimize referral flows and avoid transitions of care, can be crucial in reducing the time to initiation of breast cancer treatment.

**Perspectives:**

It is essential to improve the workflows at the different stages of health care to ensure timely initiation of oncological treatment.

## RESUMEN

**Objetivos:**

Determinar los retrasos en el inicio del tratamiento de cáncer de mama en un centro de referencia en oncología de Juiz de Fora, MG, entre 2010 y 2019.

**Métodos:**

Se trata de una cohorte con datos del Registro Hospitalario de Cáncer. La probabilidad de no iniciar tratamiento dentro de los 60 días, según la legislación brasileña, fue estimada mediante el método de Kaplan-Meier, y su asociación con los factores estudiados mediante el modelo de Cox, con la presentación del Hazard Ratios (HR) y los respectivos intervalos de confianza de 95% (IC_95_%).

**Resultados:**

Se evaluaron 911 participantes, la probabilidad de iniciar el tratamiento con retraso fue del 18,8% (IC_95_% 16,4;21,5). Quienes se sometieron a tratamiento en un servicio de salud distinto al que estableció el diagnóstico de cáncer tuvieron un riesgo significativamente mayor (HR: 3,49; IC_95_% 3,00;4,07).

**Conclusión:**

La realización del diagnóstico y tratamiento en una misma institución puede contribuir a reducir el tiempo de espera para el inicio del tratamiento.

## INTRODUCTION

Cancer stands out due to its increasing incidence, being one of the most significant public health problems today.^1^ Among women, the most common cancer globally is breast cancer, and according to the International Agency for Research on Cancer, 2,261,419 new cases and 684,996 deaths from breast cancer were estimated in the female population worldwide.^2^

In Brazil, the estimate for the three-year period from 2023 to 2025, conducted by the Instituto Nacional de Câncer (INCA),^3^ indicates that breast cancer is the most common type of cancer among women, excluding non-melanoma skin cancer, with an estimated 74,000 new cases annually.

Despite the progressive development and the implementation of public policies aimed at reducing breast cancer mortality in the country, and the fact that some advances have already been achieved, mortality still remains high.^4^ The challenge is to ensure equitable and comprehensive access to diagnosis and treatment of the disease^5^ in a context of limited resources and an increasing number of cases, which vary across different regions of Brazil.^3^

Delay between diagnosis and initiation of treatment can lead to tumor progression and consequently poor prognosis, worsening the user’s clinical condition and heir quality of life, sometimes making it irreversible.^6,7^ There is also an association between delay in diagnosis and treatment with worse disease-free survival, occurrence of lymph node metastasis, and progressive increase in tumor size, leading to advanced stage.^7^

In Brazil, Law No. 12,732/2012 guarantees the right to start cancer treatment within 60 days after the diagnosis is confirmed.^8^.^9^ However, studies show that both individual characteristics of women and the structure of healthcare services can impact the time to treatment initiation.^6,10^ Therefore, understanding potential barriers in healthcare in order to mitigate them can be crucial for improving oncological care and, consequently achieving successful treatment outcomes.

Given the lack of studies on this topic in the state of Minas Gerais, this study aims to analyze factors associated with delayed initiation of breast cancer treatment​ at an oncology referral center in the city of Juiz de Fora, between 2010 and 2019.

## METHODS

This was a retrospective hospital-based cohort study. Secondary data from the Hospital-Based Cancer Registry (*Registro Hospitalar de Câncer* - RHC) of the Hospital Maria José Baeta Reis, within the Associação Feminina de Prevenção e Combate ao Câncer (ASCOMCER), located in the city of Juiz de Fora, Minas Gerais state, were used. ASCOMCER provides 94% of its services to users of the Brazilian National Health System (*Sistema Único de Saúde* - SUS), with the remaining services being private or covered by health insurance plans. It is a High Complexity Healthcare Unit, serving as a referral center for the municipality and surrounding cities, which constitute the Immediate Geographic Region of Juiz de Fora.

The study population was comprised of female participants aged 18 years and older with diagnosis of primary malignant neoplasm of breast, who had not received prior oncological treatment, and whose treatment planning and performance were conducted at ASCOMCER. These participants were registered with the institutional RHC and had their first medical consultation between January 2010 and December 2019. The database containing the records from RHC-ASCOMCER cases was obtained in January 2022.

The variables studied included: race/skin color (White, mixed-race/Black/ Indigenous), age at diagnosis (in years: 18-49; 50-69; 70 and older), level of education (up to incomplete elementary education [including those without education]; complete elementary education; complete high school; complete or incomplete higher education), referral origin (SUS, not from SUS), medical specialty of the first treatment at the institution (surgical oncology, clinical oncology, or radiotherapy), transitions of care (categorized as “no” for people who received both diagnosis and treatment at ASCOMCER, and “yes” if the diagnosis was made at an institution other than ASCOMCER), staging (0-IIA; IIB-IIIC; IV), time of diagnosis before or after Law No. 12,732/2012 came into force. Time between diagnosis (date of anatomopathological confirmation of the tumor) and the beginning of breast cancer treatment was calculated. This variable was dichotomized with “no delay” defined as a period of 60 days or less, and “with delay” when the period was greater than 60 days, serving as the outcome variable.

Absolute and relative frequencies were calculated for categorical variables regarding participants’ sociodemographic and clinical information.

Pearson’s chi-square test was used to analyze the difference between the characteristics of those who started treatment with delay or no delay. 

As the study period included the publication date of Law No. 12,732/2012, which came into force in May 2013, we also assessed whether the proportion of participants experiencing delay in starting treatment was different in the periods before and after the law came into force. Therefore, a 5% significance level was used.

The median time and 95% confidence interval (95%CI) between diagnosis and treatment initiation was calculated using survival analysis techniques.^11^ The Kaplan-Meier method was used to estimate the probability of not starting treatment within 60 days after diagnosis. In order to apply this method, treatment initiation was considered a failure and there was no censoring, given that all participants started treatment.

Probabilities and survival curves were estimated for each of the previously described sociodemographic and clinical variables. The curves were evaluated by means of visual inspection and the log-rank test was used to determine differences between the curves.

The semiparametric Cox model was used to estimate the risk of not starting the treatment within 60 days, by calculating *hazard ratios* (HR) and their respective 95% confidence intervals (95%CI). Variables with a p-value < 0.20 in the log-rank test or chi-square test were included in the multivariable model and retained in the final model regardless of statistical significance. The proportionality of risks was assessed using Schoenfeld residuals, and the proportionality assumption was not violated. Hence, Cox models stratified by the variable “time of diagnosis (before or after the law)” were also calculated and the universal likelihood ratio test was carried out for each model. All statistical analyses were performed using the R software v.4.2.1.^12^

The study complied with the relevant resolutions and guidelines for research involving human beings, based on the National Health Council Resolution 466/2012, and was approved by the INCA Research Ethics Committee, under opinion number 5,190,175 on December 20, 2021; Certificate of Submission for Ethical Appraisal: 53090321.40000.5274.

## RESULTS

Of the 997 case records in the initial database, it could be seen that 86 (8.6%) did not meet the eligibility criteria: 47 had more than one primary tumor, 17 refused treatment, 11 had undergone prior treatment, 7 were male and 4 had no information on the initial treatment. Thus, the study population was comprised of 911 female participants.

Among the 911 female participants with breast cancer and undergoing treatment at ASCOMCER, from 2010 to 2019, 18.8% showed a time interval between diagnosis and treatment initiation greater than 60 days. There was a predominance of White people (63.6%), aged 50 to 69 years (46.9%) and with up to complete elementary education (64.6%). The majority of people (94.0%) receiving care were referred by the SUS units. The predominant medical specialty for the first treatment was surgical oncology (65.1%). Most of them (64.4%) arrived at the institution without diagnosis or treatment, i.e., there was no transition in care, they received diagnosis and treatment at the same health service. The staging distribution was 47.6% of cases in stages 0, I and IIA, 38.4% in the stages IIB-IIIC, and 14.0% in stage IV (Table 1).

**Table 1:** Sociodemographic and clinical characteristics of people with breast cancer included in the study, Juiz de Fora, Minas Gerais state, Brazil, 2010-2019

Variables	Total		Time between diagnosis and start of treatment				p-value^a^
			No delay (≤ 60 days)		With delay (> 60 days)		
	N	%	N	%	N	%	
**Total**	911	100	740	81.2	171	18.8	-
**Race/skin color** ^b^							
White	572	63.6	466	63.7	106	62.7	0.872
Mixed-race/Black/Indigenous	328	36.4	265	36.3	63	37.3	
**Age at diagnosis (years)**							
18-49	298	32.7	255	34.5	43	25.1	0.063
50-69	427	46.9	339	45.8	88	51.5	
70 and older	186	20.4	146	19.7	40	23.4	
**Education level** ^b^							
Up to incomplete elementary education	466	53.2	386	54.1	80	49.1	0.521
Complete elementary education	100	11.4	78	10.9	22	13.5	
Complete high school	232	26.5	184	25.8	48	29.4	
Complete or incomplete higher education	78	8.9	65	9.1	13	8.0	
**Referral origin**							
Brazilian National Health System (SUS)	856	94.0	691	93.4	165	96.5	0.173
Not from SUS	55	6.0	49	6.6	6	3.5	
**Medical specialty of first treatment**							
Surgical oncology	593	65.1	531	71.8	62	36.3	< 0.001
Clinical oncology	287	31.5	188	25.4	99	57.9	
Radiotherapy	31	3.4	21	2.8	10	5.8	
**Transition of care**							
No	587	64.4	550	74.3	37	21.6	< 0.001
Yes	324	35.6	190	25.7	134	78.4	
**Staging** ^b^							
0-IIA	405	47.6	340	49.3	65	40.4	0.114
IIB-IIIC	326	38.4	257	37.3	69	42.9	
IV	119	14.0	92	13.4	27	16.8	
**Time of diagnosis** ^c^							
Before the law came into force	332	36.4	291	39.3	41	24.0	< 0.001
After the law came into force	579	63.6	449	60.7	130	76.0	

a) Pearson’s chi-square test; b) Variable with missing data; c) Law No. 12,732/12 came into force on 22/05/2013.

The median time between diagnosis and treatment initiation was 9 days (95%CI 1;23) for the period from 2010 to 2019. Analyzing only the period after the law came into force, the median time was 29 days (95%CI 23;34) (data not shown in table). The proportion of participants who started treatment within 60 days after diagnosis was 81.2%. A statistically significant association (p < 0.05) between delayed treatment initiation and the medical specialty of first treatment, transition of care and time of diagnosis (Table 1) was observed.

**Figure 1:**
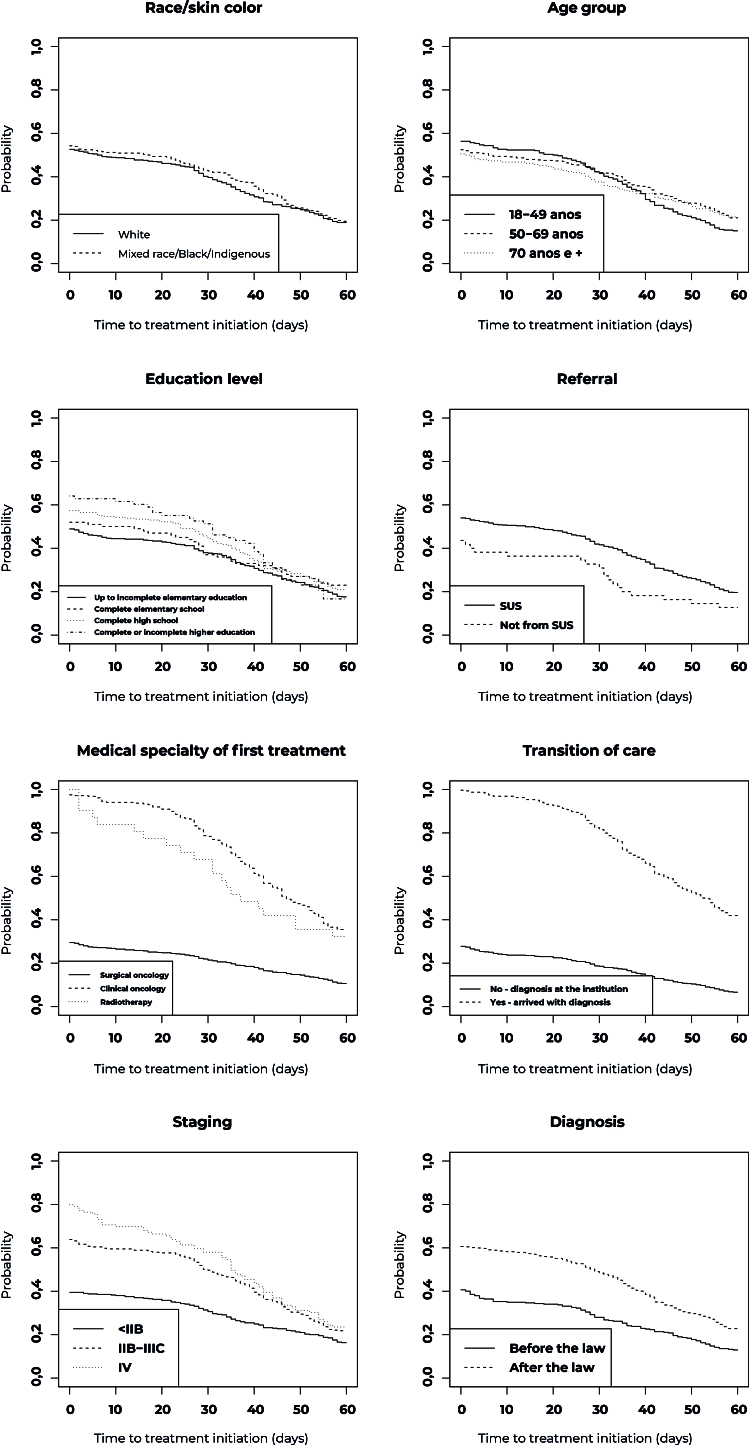
Probability curves of not starting breast cancer treatment within 60 days after diagnosis, according to sociodemographic and clinical characteristics, Juiz de Fora, Minas Gerais state, Brazil, 2010-2019

Participants referred by the SUS units and those who were diagnosed outside ASCOMCER, had higher probability of not starting treatment within 60 days compared to those who were not referred by the SUS and those who received both diagnosis and treatment at same institution (Table 2; Figure 1). There was a higher probability for people whose first treatment was in clinical oncology and radiotherapy, when compared to those who initially received treatment in oncology surgery, as well as for those with more advanced stage of breast cancer. For those diagnosed after the law came into force, the probability of not starting the first treatment within 60 days was 23.0%, while for those diagnosed before the law this probability was lower (11.1%).

**Table 2:** Probability and 95% confidence interval (95% CI) of not starting breast cancer treatment within 60 days after diagnosis, Juiz de Fora, Minas Gerais state, Brazil, 2010-2019

**Variables**	**Probability**		**p-value^a^**
	%	95%CI	
**Total**	18.8	16.4;21.5	
**Race/skin color** ^b^			
White	18.5	15.6;22.0	0.500
Mixed-race/Black/Indigenous	19.2	15.4;24.0	
**Age at diagnosis (years)**			
18-49	14.4	10.9;19.0	
50-69	20.6	17.1;24.8	0.300
70 and older	21.5	16.3;28.3	
**Education level** ^b^			
Up to incomplete elementary education	17.2	14.1;21.0	
Complete elementary education	22.0	15.2;31.8	0.500
Complete high school	20.7	16.1;26.6	
Complete or incomplete higher education	16.7	10.1;27.4	
**Referral origin**			
Brazilian National Health System (SUS)	19.3	16.8;22.1	0.040
Not from SUS	10.9	5.1;23.2	
**Medical specialty of first treatment**			
Surgical oncology	10.5	8.3;13.2	
Clinical oncology	34.5	29.4;40.5	< 0.001
Radiotherapy	32.3	19.4;53.7	
**Transition of care**			
No	6.3	4.6;8.6	< 0.001
Yes	41.4	36.3;47.1	
**Staging** ^b^			
0-IIA	16.0	12.8;20.1	
IIB-IIIC	21.2	17.2;26.1	< 0.001
IV	22.7	16.3;31.6	
**Time of diagnosisc**			
Before the law came into force	11.1	8.2;15.2	< 0.001
After the law came into force	23.0	19.8;26.6	

a) Log-rank test; a) Pearson’s Chi-square test; c) Law No. 12.732/12 came into force on 22/05/2013.

For both participants diagnosed before the law and those diagnosed after the law came into force, the medical specialty of the first treatment and the transition of care were characteristics that showed a statistically significant association with the risk of not starting the first cancer treatment within 60 days. Thus, for people diagnosed after the law, the risk was 2.29 (95%CI 0.97; 5.39) for those whose first treatment was in clinical oncology and 2.32 (95%CI 1.00;5.40) for those whose first treatment was in radiotherapy, when compared to those who started treatment in surgical oncology. In addition, participants who arrived at the institution with a diagnosis had a higher risk (HR: 3.03; 95%CI 1.59;5.78) of not starting treatment within the legally stipulated time compared to those diagnosed at the institution, i.e., when there was no transition of care (Table 3). The p-value of the universal likelihood ratio test for each model was <0.001 (data not shown in table).

**Table 3:** Adjusted Hazard Ratios (HR) and 95% confidence interval (95%CI), of not starting treatment for breast cancer within 60 days after diagnosis, Juiz de Fora, Minas Gerais state, Brazil, 2010-2019

Variables	Total				Diagnosis period							
					Before the law					After the law		
	HR	(95%CI)	p-value ^a^		HR	(95%CI)		p-value ^a^		HR	(95%CI)	p-value^a^
**Age at diagnosis (years)**												
18-49	1.00	-	0.114		1.00	-		0.220		1.00	-	0.230
50-69	1.04	(0.89;1.21)			1.06	(0.17;6.72)				1.00	(0.14;7.09)	
70 and older	0.90	(0.74;1.10)			1.03	(0.15;6.90)				0.86	(0.09;8.43)	
**Referral origin**												
Brazilian National Health System (SUS)	1.00	-	0.068		1.00	-		0.010		1.00	-	0.640
Not from SUS	0.81	(0.59;1.13)			0.82	(0.08;8.94)				0.79	(0.06;9.51)	
**Medical specialty of the first treatment**												
Surgical oncology	1.00	-	<0.001		1.00	-		<0.001		1.00	-	<0.001
Clinical oncology	2.48	(2.10;2.92)			3.02	(1.58;5.78)				2.29	(0.97;5.39)	
Radiotherapy	2.15	(1.46;3.17)			0.99	(0.14;7.19)				2.32	(1.00;5.40)	
**Transition of care**												
No	1.00	-	<0.001		1.00	-		<0.001		1.00	-	<0.001
Yes	3.49	(3.00;4.07)			4.94	(3.32;7.35)				3.03	(1.59;5.78)	
**Staging** ^b^												
0-IIA	1.00	-	<0.001		1.00	-		0.050		1.00	-	<0.001
IIB-IIIC	1.13	(0.96;1.32)			1.18	(0.22;6.22)				1.08	(0.18;6.61)	
IV	1.00	(0.80;1.24)			1.06	(0.17;6.73)				0.98	(0.13;7.27)	
**Time of diagnosis** ^c^												
Before the law came into force	1.00	-	0.129		-					-		
After the law came into force	1.31	(1.13;1.51)			-					-		

a) Global chi-square test of the variable; b) Variable with missing data; c) Law No. 12,732/12 what raisin The prevail in 05/22/2013.

## DISCUSSION

Four out of five people with breast cancer included in this study between 2010 and 2019 started treatment within 60 days after diagnosis, as mandated by law; however, a significant portion still experienced delay in initiating treatment. Those who underwent treatment at a health service other than the one where the cancer diagnosis was made had the highest risk of treatment delay. It is common knowledge that early-stage breast cancer, when diagnosed and treated promptly, has a favorable prognosis, with a five-year survival rate of up 85%.^13^

Results regarding delay in treatment initiation vary across different regions of the country. A study conducted at a referral center in the municipality of Rio de Janeiro, state of Rio de Janeiro, found that among participants whose diagnosis date was after the screening date, 83.5% started treatment within 60 days after enrollment.^14^

Another study conducted the state of Minas Gerais, involving women diagnosed with breast cancer between 2014 and 2016, and receiving care in both public and private networks, showed that 80.5% started treatment within 60 days.^15^ Similarly, in São Paulo, a study involving people aged 60 years and older diagnosed with breast cancer between 2001 and 2006, found that over 80% had a time interval of up to two months between diagnosis and treatment.^16^ On the other hand, a study using data from women with breast cancer receiving care at two referral hospital institutions in the state of Piauí, diagnosed between 2016 and 2017, revealed that the cancer care network was not enabling treatment within the time provided for in the “60-day law” for 71.6% of people with breast cancer taking part in the study.^17^

This study found that participants with early-stage breast cancer were less likely to experience delay in initiating treatment within 60 days. Although some studies have found similar results,^18,19^ others have observed that more advanced stages are associated with shorter waiting times for the treatment.^17,20^

Although some studies suggests that sociodemographic characteristics of users are factors associated with the delay in starting treatment,^120,16,21^ this study did not find evidence to support this, given that people of different race/skin color, age groups and education levels did not show differences in the percentages of delay in starting treatment.

It could be seen that, among the participants receiving care at the institution between 2010 and 2019, over half of them were outside the age group recommended by the Ministry of Health for breast cancer screening with mammography (50-69 years). Undergoing screening tests is strongly dependent on individual decisions and healthcare professionals, who must consider personal history and symptoms.^22^

It has been noted that the majority of people with breast cancer taking part in this study began their treatment with surgical oncology. This finding is consistent with that of another study that used data from 137,593 women diagnosed in 239 hospitals across Brazil between 2000 and 2011,^20^ whose percentage of those who underwent surgery as their first treatment was 67.2%.

Efforts are needed to reduce the time required to schedule the first medical oncology appointment after diagnosis in order to speed up the start of treatment. The type of first treatment may also have an impact on the time since diagnosis. For example, chemotherapy neoadjuvant can require a greater number of medical visits before therapy effectively begins.^23^ In this study, it was observed that the participants who were first treated at surgical oncology showed lower percentages of delay. In some cases, when biopsy is performed for diagnostic confirmation, the complete removal of the suspicious lesion may occur, which is then considered the first treatment.

During the period analyzed, the majority of participants arrived at the institution without diagnosis or previous treatment. Transition of care was also identified in other studies as crucial for reducing the time to treatment initiation for both breast cancer and others types of cancer. ^24,25^ A study using consolidated national data from the SUS for the 2019-2020 biennium showed that women who underwent treatment in their municipality of residence had lower rates of delay, when compared to those who underwent treatment in more distant locations.^26^

The main limitation is related to the nature of data, since this is a study using secondary data, restricting the analysis to the available information and its quality. However, it is worth highlighting that, when evaluating the quality of the RHC database, all the variables analyzed showed good or excellent quality according to criteria established in the literature.^27^ Although this study cannot be generalized to others healthcare institutions, the same methodology can to be applied to other contexts for comparison and better understanding of factors associated with delayed initiation of breast cancer treatment. Potential interactions between variables, such as medical specialty of first treatment and staging or transition of care were not assessed but could be mutually associated.

Studies evaluating the time interval between diagnosis and the initiation of treatment are essential for guiding effective policies, as they can highlight issues for improvements in health care. In conclusion, this study showed that the proportion of people who started treatment more than 60 days after diagnosis did not decrease in the period after the “60-day law came into force”, and that receiving diagnosis and treatment at same medical institution can contribute to reducing the waiting time for treatment initiation. This underscores that merely having legislation that ensures timely treatment for people with breast neoplasms does not guarantee its implementation, highlighting the need for enhanced healthcare networks and strengthened management mechanisms.
