# Supplementary material for: Factors associated with delayed initiation of breast cancer treatment at an oncology referral center in Juiz de Fora, Minas Gerais state, from 2010 to 2019: a cohort study
Source: Epidemiol Serv Saude. 2024 Aug 23;33:e20231177. doi: 10.1590/S2237-96222024v33e20231177.en (PMC11346680; doi:10.1590/S2237-96222024v33e20231177.en)
Supplement: Translation

# Translation: Fatores associados ao atraso para o início do tratamento para câncer de mama em um centro oncológico de referência em Juiz de Fora, de 2010 a 2019: estudo de coorte

**DOI:** 10.1590/S2237-96222024v33e20231177.pt

**Authors:** Fernanda de Paula Ciribelli da Silva, Mirian Carvalho Souza, Neilane Bertoni

**Affiliations:** 1 Associação Feminina de Prevenção e Combate ao Câncer de Juiz de Fora, Juiz de Fora, MG, Brasil; 2 Instituto Nacional de Câncer, Programa de Pós-Graduação em Saúde Coletiva e Controle do Câncer, Rio de Janeiro, RJ, Brasil

**Keywords:** Câncer de Mama, Tempo para Início do Tratamento, Saúde Pública, Estudo Observacional, Estudos de Coortes

## Abstract

**Objetivos:**

Analisar fatores associados ao atraso para o início do tratamento do câncer de mama em um centro de referência em oncologia em Juiz de Fora, MG, entre 2010 e 2019.

**Métodos:**

Trata-se de coorte com dados do Registro Hospitalar de Câncer. A probabilidade de não iniciar o tratamento em até 60 dias, conforme legislação brasileira, foi estimada pelo método de Kaplan-Meier, e sua associação com os fatores estudados pelo modelo de Cox, com apresentação das *hazard ratios* (HR) e respectivos intervalos de confiança de 95% (IC_95_%).

**Resultados:**

Entre as 911 participantes, a probabilidade de iniciar o tratamento com atraso foi de 18,8% (IC_95_% 16,4;21,5). Aquelas que realizaram o tratamento em um serviço de saúde diferente do que estabeleceu o diagnóstico de câncer apresentaram risco significativamente maior (HR: 3,49; IC_95_% 3,00;4,07).

**Conclusão:**

Realizar diagnóstico e tratamento na mesma instituição pode contribuir para a redução do tempo de espera para o início do tratamento.

## Contribuições do estudo

**Principais resultados:**

A probabilidade de as participantes do estudo não iniciarem o tratamento em até 60 dias foi de 18,8%. Realizar tratamento em serviço de saúde diferente do qual teve o diagnóstico foi o principal fator associado ao atraso.

**Implicações para os serviços:**

Organizar os serviços de atenção à saúde com base em estratégias que otimizem os fluxos de encaminhamento, evitando a transição no cuidado, pode ser determinante para a redução de tempo para o início do tratamento de câncer de mama.

**Perspectivas:**

É imprescindível o aprimoramento dos fluxos nas diferentes etapas na atenção em saúde, para que o tratamento oncológico seja iniciado em momento oportuno.

## INTRODUÇÃO

O câncer destaca-se pela sua crescente incidência, sendo um dos mais relevantes problemas de saúde pública da atualidade.^1^ Entre mulheres, o câncer mais incidente no mundo é o de mama, e, de acordo com a Agência Internacional de Pesquisa sobre o Câncer, para 2020, foram estimados 2.261.419 casos novos e 684.996 óbitos por câncer de mama na população feminina mundial.^2^

No Brasil, a estimativa para o triênio de 2023 a 2025, realizada pelo Instituto Nacional de Câncer (INCA),^3^ aponta o câncer de mama como o tipo mais frequente nas mulheres, excluindo o câncer de pele não melanoma, com uma estimativa de cerca de 74 mil novos casos anualmente.

Apesar do desenvolvimento e da implantação de políticas públicas de forma progressiva, visando à diminuição da mortalidade por câncer de mama no país, e de alguns avanços já terem sido obtidos, a mortalidade ainda permanece elevada.^4^ O desafio é garantir acesso equitativo e integral ao diagnóstico e ao tratamento da doença^5^ em um cenário de recursos limitados e crescente número de casos, que se dá de maneira distinta pelo território brasileiro.^3^

O atraso entre o diagnóstico e o início do tratamento pode levar ao avanço do tumor e consequentemente a um mau prognóstico, agravando o estado clínico da usuária e sua qualidade de vida, tornando-o por vezes irreversível.^6,7^ Existe também associação entre atraso no diagnóstico e no tratamento associado a pior sobrevida livre da doença, com ocorrência de metástase de linfonodos e aumento progressivo do tamanho do tumor e consequentemente estádio avançado.^7^

No Brasil, a Lei nº 12.732/2012 assegura o direito ao início do tratamento oncológico em um período igual ou inferior a 60 dias após confirmado o diagnóstico.^8,9^ Contudo, estudos apontam que tanto características individuais das mulheres quanto da estrutura dos serviços de saúde podem impactar no tempo para o início do tratamento.^6,10^ Dessa forma, compreender potenciais obstáculos na linha de cuidado à saúde, com o intuito de mitigá-los, pode ser determinante para melhorias na assistência oncológica e consequentemente no acesso a um tratamento bem-sucedido. 

Dada a ausência de estudos nessa temática no estado de Minas Gerais, o presente estudo tem como objetivo analisar fatores associados ao atraso para o início do tratamento do câncer de mama, em um centro de referência em oncologia da cidade de Juiz de Fora, entre 2010 e 2019.

## MÉTODOS

Trata-se de um estudo de coorte retrospectiva de base hospitalar. Foram utilizados dados secundários provenientes do Registro Hospitalar de Câncer (RHC) do Hospital Maria José Baeta Reis, da Associação Feminina de Prevenção e Combate ao Câncer (ASCOMCER), localizado na cidade de Juiz de Fora, MG, obtido localmente. A ASCOMCER tem 94% de seu atendimento direcionado a usuários do Sistema Único de Saúde (SUS), sendo os demais atendimentos particulares ou por planos de saúde. Trata-se de uma Unidade de Assistência de Alta Complexidade, referência para o município e cidades circunvizinhas, que compõem a Região Geográfica Imediata de Juiz de Fora.

A população do estudo compreendeu participantes do sexo feminino maiores de 18 anos de idade com diagnóstico de neoplasia maligna primária de mama, sem tratamento oncológico prévio, cujo planejamento e tratamento foram realizados na ASCOMCER, cadastradas no RHC institucional e com a primeira consulta no período entre janeiro de 2010 e dezembro de 2019. Obteve-se o banco de dados contendo os registros dos casos do RHC-ASCOMCER em janeiro de 2022.

As variáveis em estudo foram: raça/cor da pele (branca, parda/preta/indígena), idade no momento do diagnóstico (em anos: 18-49; 50-69; 70 ou mais), escolaridade (até ensino fundamental incompleto, que inclui também pessoas sem instrução; ensino fundamental completo; ensino médio completo; superior completo ou incompleto), origem do encaminhamento (SUS, não SUS), clínica do primeiro tratamento na instituição (oncologia cirúrgica, clínica ou radioterapia), transição no cuidado (sendo “não” para pessoas que realizaram diagnóstico e tratamento na ASCOMCER, e “sim” caso o diagnóstico tenha sido realizado em outra instituição que não a ASCOMCER), estadiamento (0-IIA; IIB-IIIC; IV), momento do diagnóstico antes ou depois da entrada em vigor da Lei nº 12.732/2012. Calculou-se o tempo entre o diagnóstico (data da confirmação anatomopatológica do tumor) e o início do tratamento do câncer de mama. Essa variável foi dicotomizada, sendo considerado “sem atraso” o período menor ou igual a 60 dias, e “com atraso” quando superior a 60 dias, sendo essa a variável desfecho. 

Foram calculadas frequências absolutas e relativas para variáveis categóricas sobre informações sociodemográficas e clínicas das participantes.

Foi utilizado o teste qui-quadrado de Pearson para analisar a diferença entre as características das que iniciaram o tratamento com ou sem atraso. 

Como o período do estudo compreendeu a data de publicação da Lei nº 12.732/2012, que entrou em vigor a partir de maio de 2013, avaliou-se também se a proporção de participantes com atraso para o início do tratamento foi diferente nos períodos antes e depois da entrada em vigor da referida lei. Para tal, considerou-se o nível de significância de 5%.

O tempo mediano e o intervalo de confiança de 95% (IC_95_%) entre o diagnóstico e o início do tratamento foram calculados a partir de técnicas de análise de sobrevida.^11^ Para estimar a probabilidade de não iniciar o tratamento em até 60 dias após o diagnóstico, foi utilizado o método de Kaplan-Meier. Para aplicação do método, o início do tratamento foi considerado falha e não houve censuras, visto que todas as participantes iniciaram o tratamento.

As probabilidades e as curvas de sobrevida foram estimadas para cada uma das variáveis sociodemográficas e clínicas descritas anteriormente. As curvas foram avaliadas por inspeção visual e realizou-se o teste de log-rank para determinar as diferenças entre as curvas. 

O modelo semiparamétrico de Cox foi utilizado para estimar o risco de não iniciar o tratamento em até 60 dias, por meio do cálculo das *hazard ratios* (HR) e respectivos intervalos de confiança de 95% (IC_95_%). Foram incluídas no modelo multivariável as variáveis que apresentaram p-valor < 0,20 no teste log-rank ou teste qui-quadrado, e mantidas no modelo final independentemente da significância estatística. A proporcionalidade de riscos foi avaliada a partir dos resíduos de Schoenfeld, e não se observou o pressuposto de proporcionalidade. Dessa forma, foram calculados também modelos de Cox estratificados pela variável “momento do diagnóstico (antes ou após a lei)” e realizado o teste de verossimilhança global para cada modelo. Todas as análises estatísticas foram realizadas com o uso do *software* R v.4.2.1.^12^

O estudo seguiu as resoluções e normas pertinentes referentes à pesquisa com seres humanos, com base na Resolução do Conselho Nacional de Saúde 466/2012, tendo sido aprovado pelo Comitê de Ética em Pesquisa do INCA, sob parecer de número 5.190.175 em 20 de dezembro de 2021; certificado de apresentação para apreciação ética: 53090321.40000.5274.

## RESULTADOS

Dos 997 registros de casos contidos no banco de dados inicial, observou-se que 86 (8,6%) não preenchiam de fato os critérios de elegibilidade: 47 tinham mais de um tumor primário, 17 recusaram tratamento, 11 haviam realizado tratamento anterior, 7 eram pessoas do sexo masculino e 4 não tinham informações sobre o primeiro tratamento realizado. Assim, a população de estudo compreendeu 911 participantes do sexo feminino.

Das 911 participantes com câncer de mama atendidas na ASCOMCER, no período de 2010 a 2019, 18,8% apresentaram intervalo de tempo entre diagnóstico e início de tratamento superior a 60 dias. Houve predomínio de pessoas brancas (63,6%), entre 50 e 69 anos (46,9%) e com até o ensino fundamental completo (64,6%). A maior parte (94,0%) das pessoas atendidas foram encaminhadas por unidades do SUS. A clínica predominante do primeiro tratamento foi a cirúrgica (65,1%). A maioria (64,4%) chegou à instituição sem diagnóstico e sem tratamento, ou seja, não houve transição no cuidado, tendo sido o diagnóstico e o tratamento realizados no mesmo serviço de saúde. A distribuição por estadiamento foi de 47,6% dos casos dos estádios 0, I e IIA, 38,4% nos estádios IIB-IIIC e 14,0% já no estádio IV (Tabela 1).

**Tabela 1:** Características sociodemográficas e clínicas de pessoas com câncer de mama incluídas no estudo, Juiz de Fora, MG, 2010-2019

**Variáveis**	**Total**		**Tempo entre diagnóstico e início do tratamento**				p-valor^a^
			Sem atraso (≤ 60 dias)		Com atraso (> 60 dias)		
	n	%	n	%	N	%	
**Total**	911	100	740	81,2	171	18,8	**–**
**Raça/cor da pele** ^b^							
Branca	572	63,6	466	63,7	106	62,7	0,872
Parda/preta/indígena	328	36,4	265	36,3	63	37,3	
**Idade no diagnóstico (anos)**							
18-49	298	32,7	255	34,5	43	25,1	0,063
50-69	427	46,9	339	45,8	88	51,5	
70 ou mais	186	20,4	146	19,7	40	23,4	
**Escolaridade** ^b^							
Até o ensino fundamental incompleto	466	53,2	386	54,1	80	49,1	0,521
Ensino fundamental completo	100	11,4	78	10,9	22	13,5	
Ensino médio completo	232	26,5	184	25,8	48	29,4	
Superior completo ou incompleto	78	8,9	65	9,1	13	8,0	
**Origem do encaminhamento**							
Sistema Único de Saúde (SUS)	856	94,0	691	93,4	165	96,5	0,173
Não SUS	55	6,0	49	6,6	6	3,5	
**Clínica do primeiro tratamento**							
Oncologia cirúrgica	593	65,1	531	71,8	62	36,3	< 0,001
Oncologia clínica	287	31,5	188	25,4	99	57,9	
Radioterapia	31	3,4	21	2,8	10	5,8	
**Transição no cuidado**							
Não	587	64,4	550	74,3	37	21,6	< 0,001
Sim	324	35,6	190	25,7	134	78,4	
**Estadiamento** ^b^							
0-IIA	405	47,6	340	49,3	65	40,4	0,114
IIB-IIIC	326	38,4	257	37,3	69	42,9	
IV	119	14,0	92	13,4	27	16,8	
**Momento do diagnóstico** ^c^							
Antes da entrada em vigor da lei	332	36,4	291	39,3	41	24,0	< 0,001
Depois da entrada em vigor da lei	579	63,6	449	60,7	130	76,0	

a) Teste do qui-quadrado de Pearson; b) Variável com *missing*; c) A Lei nº 12.732/12 passou a vigorar em 22/5/2013.

O tempo mediano entre o diagnóstico e o início do tratamento foi de 9 dias (IC_95_% 1;23) para o período de 2010 a 2019. Analisando-se apenas o período após a entrada em vigor da lei, o tempo mediano foi de 29 dias (IC_95_% 23;34) (dados não mostrados em tabela). A proporção de participantes que iniciaram o tratamento até 60 dias após o diagnóstico foi de 81,2%. Observou-se associação estatisticamente significativa (p < 0,05) entre o atraso para iniciar o tratamento e a clínica do primeiro tratamento, transição no cuidado e momento do diagnóstico (Tabela 1).

Participantes que foram encaminhadas por unidades do SUS e as que realizaram diagnóstico fora da ASCOMCER tiveram maior probabilidade de não iniciar o tratamento em até 60 dias do que as que não foram encaminhadas pelo SUS e as que realizaram o diagnóstico e o tratamento na mesma instituição (Tabela 2; Figura 1). Observou-se maior probabilidade para as pessoas que tiveram o primeiro tratamento na oncologia clínica e radioterapia, quando comparadas com as que foram atendidas primeiramente na oncologia cirúrgica, e também para aquelas com estadiamentos mais avançados. Para as pessoas com diagnóstico depois da entrada em vigor da lei, a probabilidade de não iniciar o primeiro tratamento em até 60 dias foi de 23,0%, enquanto para as que tiveram o diagnóstico antes da lei essa probabilidade foi inferior (11,1%).

**Figura 1:** Curvas de probabilidade de não iniciar o tratamento para câncer de mama em até 60 dias após o diagnóstico, segundo características sociodemográficas e clínicas, Juiz de Fora, MG, 2010-2019

**Tabela 2:** Probabilidade e intervalo de confiança de 95% (IC_95_) de não iniciar o tratamento para câncer de mama em até 60 dias após o diagnóstico, Juiz de Fora, MG, 2010-2019

**Variáveis**	**Probabilidade**		**p-valor** ^a^
	%	IC _95_ %	
**Total**	**18,8**	**16,4;** **21,5**	
**Raça/cor da pele** ^b^			
Branca	18,5	15,6;22,0	0,500
Parda/preta/indígena	19,2	15,4;24,0	
**Idade no diagnóstico (anos)**			
18-49	14,4	10,9;19,0	
50-69	20,6	17,1;24,8	0,300
70 ou mais	21,5	16,3;28,3	
**Escolaridade** ^b^			
Até o ensino fundamental incompleto	17,2	14,1;21,0	
Ensino fundamental completo	22,0	15,2;31,8	0,500
Ensino médio completo	20,7	16,1;26,6	
Superior completo ou incompleto	16,7	10,1;27,4	
**Origem** **do** **encaminhamento**			
Sistema Único de Saúde (SUS)	19,3	16,8;22,1	0,040
Não SUS	10,9	5,1;23,2	
**Clínica** **do** **primeiro** **tratamento**			
Oncologia cirúrgica	10,5	8,3;13,2	
Oncologia clínica	34,5	29,4;40,5	< 0,001
Radioterapia	32,3	19,4;53,7	
**Transição no cuidado**			
Não	6,3	4,6;8,6	< 0,001
Sim	41,4	36,3;47,1	
**Estadiamento** ^b^			
0-IIA	16,0	12,8;20,1	
IIB-IIIC	21,2	17,2;26,1	< 0,001
IV	22,7	16,3;31,6	
**Momento do diagnóstico** ^c^			
Antes da entrada em vigor da lei	11,1	8,2;15,2	< 0,001
Depois da entrada em vigor da lei	23,0	19,8;26,6	

a) Teste de log-rank; b) Variável com *missing*; c) A Lei nº 12.732/12 passou a vigorar em 22/5/2013.

Tanto para as participantes com diagnóstico antes da lei quanto para as que tiveram o diagnóstico depois da entrada em vigor da lei, a clínica do primeiro tratamento e a transição no cuidado foram características que apresentaram associação estatisticamente significativa com o risco de não iniciar o primeiro tratamento para o câncer em até 60 dias. Assim, para as pessoas que tiveram diagnóstico após a lei, o risco foi de 2,29 (IC_95_% 0,97; 5,39) para as que tinham o primeiro tratamento na oncologia clínica e de 2,32 (IC_95_% 1,00;5,40) na radioterapia, quando comparadas às que iniciavam o tratamento com a cirurgia. Também, as participantes que chegavam à instituição já com um diagnóstico tinham risco maior (HR:3,03; IC_95_% 1,59;5,78) de não iniciar o tratamento no tempo previsto em lei do que as que realizavam o diagnóstico na instituição, ou seja, quando não havia transição no cuidado (Tabela 3). O p-valor do teste de verossimilhança global para cada modelo foi <0,001 (dados não mostrados em tabela).

**Tabela 3:** *Hazard ratios* (HR) ajustadas, e respectivos intervalos de confiança de 95% (IC_95_%), de não se iniciar o tratamento para câncer de mama em até 60 dias após o diagnóstico, Juiz de fora, MG, 2010-2019

**Variáveis**	**Total**			**Período do diagnóstico**						
				Antes da lei				Após a lei		
	HR	(IC _95_ % )	p-valor ^a^	HR	(IC _95_ % )		p-valor ^a^	HR	(IC _95_ % )	p-valor ^a^
Idade no diagnóstico (anos)										
18-49	1,00	-	0,114	1,00	-		0,220	1,00	-	0,230
50-69	1,04	(0,89;1,21)		1,06	(0,17;6,72)			1,00	(0,14;7,09)	
70 ou mais	0,90	(0,74;1,10)		1,03	(0,15;6,90)			0,86	(0,09;8,43)	
**Origem do encaminhamento**										
Sistema Único de Saúde (SUS)	1,00	-		1,00	-		0,010	1,00	-	0,640
Não SUS	0,81	(0,59;1,13)	0,068	0,82	(0,08;8,94)			0,79	(0,06;9,51)	
**Clínica** **do primeiro tratamento**										
Oncologia cirúrgica	1,00	-	< 0,001	1,00	-		< 0,001	1,00	-	< 0,001
Oncologia clínica	2,48	(2,10;2,92)		3,02	(1,58;5,78)			2,29	(0,97;5,39)	
Radioterapia	2,15	(1,46;3,17)		0,99	(0,14;7,19)			2,32	(1,00;5,40)	
**Transição no cuidado**										
Não	1,00	-	< 0,001	1,00	-		< 0,001	1,00	-	< 0,001
Sim	3,49	(3,00;4,07)		4,94	(3,32;7,35)			3,03	(1,59;5,78)	
**Estadiamento** ^b^										
0-IIA	1,00	-	< 0,001	1,00	-		0,050	1,00	-	< 0,001
IIB-IIIC	1,13	(0,96;1,32)		1,18	(0,22;6,22)			1,08	(0,18;6,61)	
IV	1,00	(0,80;1,24)		1,06	(0,17;6,73)			0,98	(0,13;7,27)	
**Momento do diagnóstico** ^c^										
Antes da entrada em vigor da lei	1,00	-	0,129	-				-		
Depois da entrada em vigor da lei	1,31	(1,13;1,51)		-				-		

a) Teste qui-quadrado global da variável; b) Variável possui *missing*; c) A Lei nº 12.732/12 passou a vigorar em 22/5/2013.

## DISCUSSÃO

Quatro em cinco pessoas com câncer de mama incluídas no estudo entre 2010 e 2019 iniciaram o tratamento em até 60 dias após o diagnóstico, conforme preconiza a lei; ainda permanece parcela relevante com atraso para iniciar o tratamento. Aquelas que realizaram o tratamento em um serviço de saúde diferente do que estabeleceu o diagnóstico de câncer foram as que apresentaram maior risco de iniciar o tratamento com atraso. Sabe-se que, em estádios iniciais, o câncer de mama, quando diagnosticado e tratado oportunamente, se revela um tumor de bom prognóstico, e a sobrevida em cinco anos chega a 85%.^13^

Os resultados para essa questão do atraso para o início do tratamento em outros locais do país são diversos. Em estudo realizado em um centro de referência no Rio de Janeiro, entre participantes cuja data do diagnóstico foi posterior à data da triagem, 83,5% iniciaram o tratamento em até 60 dias após a matrícula.^14^

Em outro estudo realizado no estado de Minas Gerais, com mulheres diagnosticadas com câncer de mama entre 2014 e 2016 atendidas nas redes pública e privada, 80,5% iniciaram o tratamento em até 60 dias.^15^ O resultado foi similar ao que foi encontrado em São Paulo, em estudo com pessoas com 60 anos ou mais diagnosticadas com câncer de mama entre 2001 e 2006, onde mais de 80% tiveram até dois meses de intervalo entre o diagnóstico e o tratamento.^16^ Já em um estudo realizado com dados de mulheres com câncer de mama atendidas em duas instituições hospitalares de referência no estado do Piauí, diagnosticadas entre 2016 e 2017, evidenciou-se que a rede de atenção oncológica não estava possibilitando o tratamento no tempo previsto na “lei dos 60 dias” para 71,6% das pessoas com câncer de mama do estudo.^17^

Encontrou-se neste estudo que as participantes com câncer de mama em estádios iniciais apresentaram menor probabilidade de não iniciar o tratamento no prazo de 60 dias. Embora alguns estudos tenham encontrado resultados similares,^18,19^ outros observam que estádios mais avançados estariam associados a menor tempo de espera para o tratamento.^17,20^

Embora alguns estudos apontem características sociodemográficas das usuárias como sendo fatores associados ao atraso para o início do tratamento,^10,16,21^ no presente estudo não se evidenciou esse fato, de forma que pessoas de diferentes raça/cor da pele, faixas etárias e graus de escolaridade não apresentaram diferenças nos percentuais de atraso para iniciar o tratamento.

Observou-se que, entre as participantes atendidas na instituição no período de 2010 a 2019, mais da metade estavam fora da faixa etária preconizada pelo Ministério da Saúde para o rastreamento mamográfico do câncer de mama (50-69 anos). A realização de exames é fortemente dependente de decisões individuais e dos profissionais de saúde, que devem considerar o histórico pessoal e as queixas das pacientes.^22^

Verificou-se que a maioria das pessoas com câncer de mama participantes deste estudo iniciou o tratamento pela clínica cirúrgica. Esse achado é consoante com o encontrado em um estudo que utilizou dados de 137.593 mulheres diagnosticadas em 239 unidades hospitalares do Brasil entre 2000 e 2011,^20^ cujo percentual que recebeu cirurgia como primeiro tratamento foi de 67,2%.

São necessários esforços para que haja a redução dos tempos necessários para agendamento da primeira consulta médica oncológica, após o diagnóstico, para agilizar o início do tratamento. O tipo de primeiro tratamento também pode impactar no tempo desde o diagnóstico. A quimioterapia neoadjuvante, por exemplo, pode demandar um número maior de visitas médicas antes do início da terapia efetivamente.^23^ No presente estudo, observou-se que as participantes que eram atendidas primeiramente na oncologia cirúrgica apresentaram menores percentuais de atraso. Em alguns casos, ao se realizar a biópsia para confirmação diagnóstica, há a retirada completa da lesão suspeita, já sendo esse considerado o primeiro tratamento.

No período analisado, a maioria das participantes chegou à instituição sem diagnóstico e sem tratamento anterior. A transição no cuidado também foi apontada em outros estudos como sendo determinante para a redução de tempo para início do tratamento, tanto de câncer de mama como para outros tipos de câncer.^24,25^ Estudo realizado com dados nacionais consolidados do SUS do biênio 2019-2020 apontou que mulheres que se trataram no município de residência tiveram menor percentual de atraso em comparação com aquelas que realizaram o tratamento em locais mais distantes.^26^

A limitação principal refere-se à natureza dos dados, uma vez que se trata de um estudo com uso de dados secundários, de forma que as análises ficam restritas à informação disponível e à sua qualidade. Contudo, ressalta-se que, ao se avaliar a qualidade do banco de dados do RHC, observou-se que todas as variáveis analisadas apresentavam qualidade boa ou excelente de acordo com critério estabelecido na literatura.^27^ Embora este estudo não seja passível de generalização para outras instituições de saúde, a mesma metodologia pode ser aplicada a outras realidades para fins de comparação e melhor entendimento dos fatores associados ao atraso para início do tratamento de câncer de mama. Não foram avaliadas possíveis interações entre variáveis, como, por exemplo, clínica do primeiro tratamento e estadiamento ou transição do cuidado, que poderiam estar mutuamente associadas.

Estudos que avaliam o intervalo de tempo entre o diagnóstico e o início de tratamento são necessários para direcionar políticas resolutivas, na medida em que podem apontar questões para melhorias na assistência em saúde. Em conclusão, este estudo apontou que a proporção de pessoas que iniciaram o tratamento com mais de 60 dias após o diagnóstico não se reduziu no período após a vigência da “lei dos 60 dias” , e que a realização do diagnóstico e do tratamento na mesma instituição médica pode contribuir para a redução do tempo de espera para o início do tratamento. Evidencia-se que o fato de haver uma legislação que versa sobre o direito das pessoas com neoplasia de realizarem o tratamento em tempo oportuno não assegura seu cumprimento, necessitando que sejam aprimoradas as redes de assistência e consolidados os mecanismos de gestão.
